# Association between failed eradication of 7-day triple therapy for *Helicobacter pylori* and untreated dental caries in Japanese adults

**DOI:** 10.1038/s41598-024-54757-8

**Published:** 2024-02-19

**Authors:** Komei Iwai, Tetsuji Azuma, Takatoshi Yonenaga, Yasuyuki Sasai, Kazutoshi Watanabe, Akihiro Obora, Fumiko Deguchi, Takao Kojima, Takaaki Tomofuji

**Affiliations:** 1 Department of Community Oral Health, School of Dentistry, Asahi University, 1851-1 Hozumi, Mizuho, Gifu 501-0296 Japan; 2 Asahi University Hospital, 3-23 Hashimoto-Cho, Gifu, Gifu 500-8523 Japan

**Keywords:** Health care, Medical research, Risk factors

## Abstract

*Helicobacter pylori* (*H. pylori*) infection is a cause of gastric disorders and is treated mainly by pharmacotherapy with antimicrobial agents. An association has been reported between dental caries and *H. pylori* infection. As antimicrobial agents are less effective inside dental caries because of impaired blood circulation, the presence of untreated dental caries (decayed teeth) may influence the success of *H. pylori* eradication treatment. In this cross-sectional study, we examined whether failed eradication of *H. pylori* was associated with decayed teeth in Japanese adults. Enrolled were 226 participants who received dental checkups among those treated for eradication of *H. pylori* at Asahi University Hospital between April 2019 and March 2021. Treatment efficacy was assessed by urea breath test. Eradication failed in 38 participants (17%), decayed teeth in 32 participants (14%), and number of 0.34 teeth per participants. Multivariate logistic regression analyses showed that failed eradication of *H. pylori* was associated with decayed teeth (presence: odds ratio, 2.672; 95% confidence interval, 1.093–6.531) after adjusting for gender, age, and brushing frequency. These results indicate that failed eradication of *H. pylori* was associated with decayed teeth and suggest that untreated dental caries may impact treatment for eradication of *H. pylori*.

## Introduction

*Helicobacter pylori* (*H. pylori*) infection causes gastric diseases such as chronic gastritis, gastric ulcer, duodenal ulcer, and gastric cancer^1–3^. It is designated as a Group 1 carcinogen by the International Agency for Research on Cancer^4^ and is reported to cause over 90% of gastric cancers^5^. In 2017, it was reported that approximately 36 million people in Japan were infected with *H. pylori*^6^, and that infection rates increased with increasing age^7^. Importantly, there were 120,000 cases of gastric cancer in Japan in 2021, which is the third highest among all cancers^8^. Therefore, control of *H. pylori* infection is very important in the field of public health in Japan.

Drug-based eradication therapy is widely used to treat *H. pylori* infection^9,10^. In most cases, those who test positive for *H. pylori* infection are treated with 7-day triple therapy^11,12^. However, this therapy is unsuccessful in some cases^13^. It has been reported that failed eradication may be due to the presence of *H. pylori* that is drug-resistant to antimicrobial agents, but much remains unclear and further research is required^13,14^.

A previous study reported the detection of the *H. pylori* gene within untreated dental caries (decayed teeth) and found a significant association between presence of this gene and *H. pylori* infection^15^. We have also reported an association between dental caries and *H. pylori* infection in a cross-sectional study^16^. Bacterial invasion and colony formation occur in the dental pulp of teeth with caries^17^. In infected teeth, biofilms formed by the colonizing bacteria provide chemical protection to microbial cells, thus reducing the efficacy of antimicrobials^18^. Therefore, *H. pylori* inside dental caries may be less susceptible to antimicrobial agents. We hypothesized that there is an association between failed eradication of *H. pylori* and the presence of dental caries. Therefore, the purpose of our study was to examine the association between failed eradication of *H. pylori* and untreated dental caries in Japanese adults.

## Results

In our study, 226 participants (150 males and 76 females, mean age 52.7 years) were included in the analysis. Table 1 lists the participants’ characteristics according to the outcome of treatment to eradicate *H. pylori*. Eradication was unsuccessful in 38 participants (17%), and significantly fewer participants in this group brushing frequency ≥ 2 times/day (n = 27) compared with those whose treatment was successful (n = 162; p = 0.022). The rate of decayed teeth was significantly higher in participants with failed eradication than in those with successful eradication (p = 0.004). There was no significant difference in the presence or absence of filled and missing teeth between participants with successful and failed eradication of *H. pylori* (p = 0.161).

**Table 1:** Participant characteristics according to the success or failure of *H. pylori* eradication treatment.

Factor		Eradication of *H. pylori*		*p-*value*
		Succeeded (n = 188)	Failed (n = 38)	
Male^a^		123 (65%)	27 (71%)	0.503
Age (years)	− 64	169 (90%)	32 (84%)	0.308
	65–	19 (10%)	6 (16%)	
Smoking habits^b^		16 (9%)	5 (13%)	0.368
Amount of drinking^c^		34 (18%)	8 (21%)	0.668
Hypertension^b^		5 (3%)	1 (3%)	0.992
Diabetes^b^		3 (2%)	1 (3%)	0.659
Heart disease^b^		8 (4%)	0 (0%)	0.195
Regular dental checkups^b^		122 (65%)	20 (53%)	0.154
Brushing frequency ≥ 2 times/day^b^		162 (86%)	27 (71%)	0.022
Gingival bleeding^b^		70 (37%)	20 (53%)	0.077
Periodontal pocket depth ≥ 4 mm^b^		116 (62%)	23 (61%)	0.892
Decayed teeth^b^		21 (11%)	11 (29%)	0.004
Filled teeth^b^		185 (98%)	36 (95%)	0.161
Missing teeth^b^		74 (39%)	20 (53%)	0.130
Number of present teeth ≥ 28 teeth^b^		127 (68%)	25 (66%)	0.833
Medication history^b^		31 (17%)	7 (18%)	0.772

*H. pylori Helicobacter pylori.*

*p < 0.05, using chi-square test.

^a^Male (proportion of male).

^b^Presence (proportion of presence).

^c^Heavy (proportion of heavy).

The results of univariate logistic regression analysis with failed eradication of *H. pylori* as the dependent variable are summarized in Table 2. The results showed a statistically significant association of failed eradication of *H. pylori* with brushing frequency ≥ 2 times/day (presence; odds ratio (OR), 0.394; 95% confidence interval (CI) 0.175–0.889) and decayed teeth (presence; OR, 3.240; 95% CI 1.406–7.468). There was no significant association of presence or absence of filled and missing teeth with successful or failed eradication of *H. pylori*.

**Table 2:** Univariate logistic regression analyses in participants with failed eradication of *H. pylori*.

Factor		Crude ORs	95% Cl	*p*-value
Gender	Female	1	(Reference)	0.504
	Male	1.297	0.605–2.781	
Age (years)	− 64	1	(Reference)	0.313
	65–	0.668	0.618–4.500	
Smoking habits	Absence	1	(Reference)	0.372
	Presence	1.629	0.558–4.753	
Amount of drinking	Not heavy	1	(Reference)	0.668
	Heavy	1.208	0.509–2.865	
Hypertension	Absence	1	(Reference)	0.992
	Presence	0.989	0.112–8.715	
Diabetes	Absence	1	(Reference)	0.662
	Presence	1.667	0.169–16.467	
Regular dental checkups	Absence	1	(Reference)	0.156
	Presence	0.601	0.297–1.215	
Brushing frequency ≥ 2 times/day	Absence	1	(Reference)	0.025
	Presence	0.394	0.175–0.889	
Gingival bleeding	Absence	1	(Reference)	0.080
	Presence	1.873	0.928–3.780	
Periodontal pocket depth ≥ 4 mm	Absence	1	(Reference)	0.892
	Presence	0.952	0.466–1.943	
Decayed teeth	Absence	1	(Reference)	0.004
	Presence	3.240	1.406–7.468	
Filled teeth	Absence	1	(Reference)	0.186
	Presence	0.292	0.047–1.810	
Missing teeth	Absence	1	(Reference)	0.133
	Presence	1.712	0.849–3.450	
Number of present teeth ≥ 28 teeth	Absence	1	(Reference)	0.833
	Presence	0.924	0.442–1.929	
Medication history	Absence	1	(Reference)	0.772
	Presence	1.144	0.462–2.830	

*H. pylori Helicobacter pylori*, *ORs* odds ratios, *CI* confidence interval.

The results of multivariate stepwise logistic regression analysis with failed eradication of *H. pylori* as the dependent variable are shown in Table 3. There was a significant association of failed eradication of *H. pylori* with the presence of decayed teeth (presence; OR 2.672; 95% CI 1.093–6.531) after adjusting for gender, age, and presence or absence of brushing frequency ≥ 2 times/day.

**Table 3:** Multivariate stepwise logistic regression analyses in participants with failed eradication of *H. pylori*.

Factor		ORs	95% Cl	*p-*value
Gender	Female	1	(Reference)	0.659
	Male	1.194	0.543–2.627	
Age (years)	− 64	1	(Reference)	0.282
	65–	1.766	0.627–4.974	
Brushing frequency ≥ 2 times/day	Absence	1	(Reference)	0.146
	Presence	0.520	0.215–1.257	
Decayed teeth	Absence	1	(Reference)	0.031
	Presence	2.672	1.093–6.531	

*H. pylori Helicobacter pylori*, *ORs* odds ratios, *CI* confidence interval.

Adjustment for gender, age, presence or absence of brushing frequency ≥ 2 times/day, and presence or absence of decayed teeth.

*p* = 0.478, using Hosmer–Lemeshow fit test.

Table 4 shows the proportions of participants with failed eradication of *H. pylori* according to the number of decayed teeth. Eradication treatment failed in 24% (5/21) of those with 1 decayed tooth, in 40% (2/5) of those with 2 decayed teeth, and in 67% (4/6) of those with ≥ 3 decayed teeth. The proportion of participants with failed eradication increased significantly with increasing number of decayed teeth (p = 0.002).

**Table 4:** Participants with failed eradication of *H. pylori* according to number of decayed teeth.

Factor	Number of decayed teeth				*p* value*
	0 (n = 194)	1 (n = 21)	2 (n = 5)	3- (n = 6)	
Failed eradication of *H. pylori*	27 (14%)	5 (24%)	2 (40%)	4 (67%)	0.002

*H. pylori Helicobacter pylori.*

*p < 0.05, using chi-square test.

## Discussion

To the best of our knowledge, this study is the first to examine the association between failed eradication of *H. pylori* and untreated dental caries in Japanese adults. The results showed that participants with failed eradication had a greater number of decayed teeth than those with successful eradication. Logistic regression analyses also revealed that failed eradication of *H. pylori* was associated with the presence or absence of decayed teeth after adjusting for gender, age, and brushing frequency ≥ 2 times/day. These results suggest that the presence of decayed teeth could be a risk factor of failed eradication of *H. pylori*. Furthermore, the rate of failed eradication of *H. pylori* increased according to the number of decayed teeth.

There are some possible mechanisms for the relationship between failed eradication of *H. pylori* and decayed teeth. A previous study reported that *H. pylori* was detected in the infected dental cavities of decayed teeth^15^. Blood circulation is poor in lesions of infected dental cavities^19^. Because antimicrobials are less likely to transfer into blood vessels in infected tissues^19^, antimicrobials may have difficulty penetrating an infected dental cavity. For these reasons, *H. pylori* adherent within an infected dental cavity might not be eradicated by antimicrobial agents. In addition, bacteria that invade decayed teeth undergo colony formation^17^. Colonized bacteria form biofilms that provide protection against chemical agents, making them less susceptible to drugs^18^. Therefore, it is also possible that *H. pylori* within decayed teeth are protected from chemical invasion in the same way as colonized bacteria, thus reducing the efficacy of antimicrobials^18^.

An infected dental cavity within a decayed tooth is difficult to reach with a brush during oral cleaning, making it difficult to remove accumulated oral bacteria^20^. Therefore, dentists treat decayed teeth by removing the infected tooth structure and filling the hole with a prosthetic material that prevents accumulation of oral bacteria on the tooth^21^. In the present study, we found no significant association of the presence or absence of filled teeth with success or failure to eradicate *H. pylori*. This result indicates that even if tooth decay has occurred, the risk of failure to eradicate *H*. pylori can be eliminated as long as the affected teeth are treated promptly.

In our study, the proportion of failed eradication of *H. pylori* among all participants was 17%, which is similar to those previously reported in studies conducted in Japan (8.9–21.2%)^22–25^. Therefore, the characteristics of the present participants are representative of the standard characteristics of Japanese. However, the external validity of our study should be considered, because all participants were recruited from Asahi University.

In our study, univariate analysis showed a significant association between eradication of *H. pylori* as a 7-day triple therapy and brushing frequency, but no association was found in multivariate analysis. Therefore, brushing frequency ≥ 2 times/day tended to be associated with successful eradication of *H. pylori* as a 7-day triple therapy. Previous study was reported that brushing frequency ≥ 2 times/day decreases dental caries experience^26^. Therefore, in our study, brushing frequency ≥ 2 times/day may also was associated with successful eradication of *H. pylori* as a 7-day triple therapy via reduction in dental caries.

We used the Hosmer–Lemeshow test to examine the goodness of fit in the multivariate logistic regression analysis model and to test whether the observed event rate in the subgroup model fit the expected event rate. In this test, a *p*-value > 0.05 is considered to indicate good fit^27^. Therefore, the present *p*-value of 0.478 indicates accurate performance of our multivariate logistic regression model.

Our study suggests that prevention of dental caries and early treatment of dental caries increases of successful eradication of *H. pylori* as a 7-day triple therapy. Therefore, as future recommendations for our study, it is important not only to instruct participants with positive for *H. pylori* test to get eradication of *H. pylori* therapy, but also to have them visit dental clinic. If the results indicate that participants have dental caries, it is important to initiate dental caries treatment before eradication of *H. pylori* therapy.

There are some limitations of our study. First, the timing of the incidence, the severity, and level of dental caries was not confirmed. The timing of the incidence of dental caries was not confirmed. In the future, we would like to investigate the effects on failed eradication of *H. pylori* of duration of time without treatment for dental caries and of the severity of dental caries. Second, the presence of *H. pylori* resistant to antimicrobials has been reported to be associated with failed eradication of *H. pylori*^13,14^. However, we did not investigate whether *H. pylori* carried by the present participants was resistant to antimicrobials. Third, this study investigated eradication of *H. pylori* using only 7-day *H. pylori* triple therapy. The drug combinations that we used for *H. pylori* eradication are those used most commonly for primary eradication of *H. pylori* in Japan^28,29^. However, the rates of eradication of *H. pylori* using drugs other than the antimicrobial agents used in the present study should be considered. Fourth, it was not possible to confirm whether all of filled teeth were caused by dental caries. It is possible that some of filled teeth were not caused by dental caries, such as post-fracture of a tooth procedures. Finally, urea breath test was used to evaluate the treatment for eradication of *H. pylori* in our study. This is because urea breath test is one of the most sensitive and specific tests for *H. pylori*^30–33^. However, in addition to urea breath test, there are other tests for *H. pylori*, including antibody assay, fecal antigen assay, rapid urease test, and histoscopic examination test^30–33^. Therefore, results may differ when these tests are used.

In conclusion, our study showed that failure to eradicate *H. pylori* was associated with the presence of decayed teeth among Japanese adults. Untreated dental caries may have an impact on failure to eradicate *H. pylori*.

## Methods

### Formulation of question

Our study was developed into a Population, Intervention, Comparison, Outcomes (PICO) format. In other words, “P” were for participants who received treatment to eradicate *H. pylori* and dental checkups, “I” were for successful eradication, “C” were for failure eradication, and “O” were for presence or absence with decayed teeth.

### Participants

In our study, 243 individuals who received treatment to eradicate *H. pylori* and dental checkups at Asahi University Hospital between April 2019 and March 2021 were participated. Excluded were participants with a medical history of gastric disease (n = 12) because of the high risk of *H. pylori* infection, and those who regularly used antibiotics (n = 5). These data were confirmed from participants' medical records and self-administered questionnaires. A final total of 226 participants (150 males and 76 females, mean age 52.7 years) were included in the analysis. The efficacy of treatment was evaluated by urea breath test. The performance of the urea breath test has been previously reported as excellent (sensitivity; 95–98%, specificity; 95–97%)^30–33^.

### Eradication treatment for *H. pylori* and evaluation of efficacy

Participants underwent treatment for eradication of *H. pylori* as a 7-day triple therapy (*Amoxicillin*; penicillin antibiotics, *Clarithromycin*; macrolide antibiotics, proton pump inhibitors) taken twice per day, after breakfast and dinner. At one month after completion, all patients underwent the urea breath test to determine whether the eradication treatment had been successful or unsuccessful^28,29^.

### Participant characteristics

Data regarding gender, age, hypertension, diabetes, heart disease, and medication history were obtained from the medical records of Asahi University Hospital.

### Smoking habits, drinking habit, and oral health

Smoking habit was defined as currently smoking at least one cigarette/day (presence or absence)^34^. Drinking habit was defined as heavy for a current alcohol consumption of ≥ 2 go/day (where “go” is a traditional Japanese unit of volume measurement, corresponding to 23 g of ethanol) (heavy or not heavy)^35^. These habits were surveyed in participants in a self-administered questionnaire. Data on the following aspects of oral health were obtained: regular dental checkups (presence or absence), brushing frequency (≥ 2 times/day or < 2 times/day), gingival bleeding (presence or absence), periodontal pocket depth (≥ 4 mm or < 4 mm), decayed teeth (presence or absence), filled teeth (presence or absence), missing teeth (presence or absence), number of teeth (≥ 28 teeth or < 28 teeth). Regular dental checkups was defined as visiting the dentist at least once every 6 months^36^. Five dentists participated in our study; any one of five dentists checked the oral status of each participant. Five dentists repeated the calibration until each dentists confirmed that agreement (kappa value) exceeded 0.8, and then the examination was performed after each dentists agreed. Gingival bleeding and periodontal pocket depth were evaluated using the coded values of the Community Periodontal Index (CPI), in which the presence of gingival bleeding is scored as 1, and periodontal pocket depth ≥ 4 mm is scored as 1 or 2^37^. Dental caries status was evaluated using decayed teeth (presence or absence) and filled teeth (presence or absence)^38^. In addition, missing teeth (presence or absence), possibly due to dental disease such as decay, periodontal disease, and trauma was also investigated^37^.

### Statistical analysis

The normality of continuous variables was confirmed using Kolmogorov–Smirnov tests. Significant differences in characteristics according to the success or failure of eradication of *H. pylori* were assessed using chi-square test and Mann–Whitney U test. Univariate and multivariate logistic regression analyses were performed with failed eradication of *H. pylori* as the dependent variable. In multivariate stepwise logistic regression analysis, variables with p > 0.05 were excluded from the model; in addition, variables that were significantly different in univariate logistic regression analysis in addition to gender and age were selected for adjustment factors. The proportion of participants with failed eradication of *H. pylori* according to the number of decayed teeth was assessed using the chi-square test. The suitability of this model was confirmed by Hosmer–Lemeshow fit test. All data were analyzed using statistical analysis software (SPSS statistics version 27; IBM Japan, Tokyo, Japan). All *p*-values < 0.05 were considered statistically significant.

### Research ethics

Our study was approved by the Asahi University Ethics Committee (No. 27010) and was conducted in accordance with the Declaration of Helsinki. All participants provided written informed consent. Our cross-sectional study was conducted following the STROBE guidelines.

## Data Availability

The data that support the findings of this study are not publicly available from Asahi University Hospital as they were used under license for the current study and availability restrictions apply. However, data are available from the authors upon reasonable request and with the permission of Asahi University Hospital.
